# Cost-effectiveness of the MitraClip device in German heart failure patients with secondary mitral regurgitation

**DOI:** 10.1007/s10198-022-01476-4

**Published:** 2022-05-27

**Authors:** Bent Estler, Volker Rudolph, Yana Seleznova, Arim Shukri, Stephanie Stock, Dirk Müller

**Affiliations:** 1 Institute for Health Economics and Clinical Epidemiology, University of Cologne, Albertus-Magnus-Platz, 50923 Cologne, Germany; 2 Heart & Diabetes Centre NRW, General and Interventional Cardiology/Angiology, University Hospital of the Ruhr University Bochum, Bad Oeynhausen, Chelsea, Germany; 3 Institute for Health Economics and Clinical Epidemiology, University of Cologne, Gleueler Str. 176-178, 50935 Cologne, Germany

**Keywords:** Heart failure, Secondary mitral regurgitation, Cost-effectiveness, Transcatheter-valve repair, MitraClip, Germany

## Abstract

**Aim:**

To evaluate the cost-effectiveness of the MitraClip device (MitraClip) in addition to optimal medical therapy (OMT) in patients with heart failure and secondary mitral regurgitation in Germany.

**Methods and results:**

A model-based economic evaluation was performed to estimate the incremental cost per quality-adjusted life-years (QALYs) for patients with a moderate-to-severe or severe secondary mitral regurgitation receiving MitraClip plus OMT compared with OMT alone from the statutory health insurance (SHI) perspective. Transition probabilities, data on survival rates, and hospitalization rates were obtained from the COAPT trial, a randomized-controlled multicenter trial. Data on health utility and costs were taken from published evidence. To assess parameter uncertainty, several deterministic and probabilistic sensitivity analyses were performed. The incremental costs per QALY gained were € 59,728 (costs/incremental life years gained: € 42,360). The results were most sensitive to the transition probabilities and the hospitalization rates. The probabilistic sensitivity analysis showed that the MitraClip strategy was cost-effective with a probability of 80% at a willingness-to-pay threshold of € 67,000/QALY.

**Conclusions:**

Depending on the willingness-to-pay threshold, for patients with heart failure and a moderate-to-severe or severe secondary mitral regurgitation the MitraClip can be cost-effective from the perspective of the German SHI.

**Graphical abstract:**

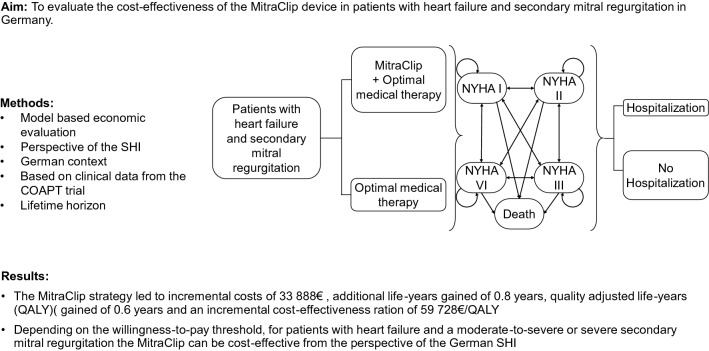

**Supplementary Information:**

The online version contains supplementary material available at 10.1007/s10198-022-01476-4.

## Introduction

Heart failure is a rapidly growing public health issue with more than 37 million persons affected worldwide [1] and is still one of the leading causes of premature death [2]. Due to its multifactorial etiology, the progression of HF increasingly affects patients’ quality of life by causing symptoms such as dyspnea, fatigue, and fluid retention [3]. For example in Germany, the prevalence of HF was 3.4%, corresponding to approximately 2.5 million affected individuals in 2017 [4].

In addition to the burden of disease, HF is estimated to incur around 1–2% of the annual healthcare budget in the Western societies [5]. Because HF is one of the leading causes of hospitalization in adults, the majority of HF treatment costs are incurred in the inpatient setting [1, 5]. Reducing admission rates due to HF is assumed to be the most promising approach to decrease the economic burden of HF [6].

Many patients with HF develop secondary mitral regurgitation which is associated with a poor prognosis (i.e., reduced life expectancy) [5, 7]. In addition to medical treatment, different minimal-invasive procedures for treating secondary mitral regurgitation such as the MitraClip were developed in the late 1990s. Compared to medical therapy alone, the MitraClip resulted in a lower rate of hospitalization due to HF and lower all-cause mortality within 24 months of follow-up [7].

In addition to the safety, clinical effectiveness and efficacy, evaluating the cost-effectiveness of new devices is an important factor in a resource-constrained healthcare system. Based on clinical data from the COAPT trial [8], a patient-level simulation showed incremental costs for the transcatheter mitral valve repair of $55,600 per quality-adjusted life-year (QALY) gained for the US-system when compared with optimal medical therapy (OMT) which is considered high economic value for cardiac therapies in the United States (US) [9].

As Germany is the second largest implanter of MitraClip devices worldwide, our analysis aims to evaluate the cost-effectiveness of the MitraClip in combination with OMT in patients with HF and secondary mitral regurgitation compared with OMT alone [10].

## Methods

**Fig. 1:**
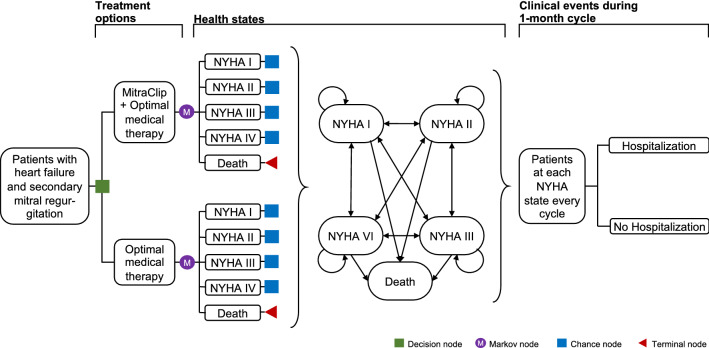
Combined model comparing the MitraClip with optimal medical therapy. The model starts with a decision tree splitting the patients into the MitraClip Group or the OMT group. The patients then enter a Markov model with five health states. These include the four NYHA classes and death. HF Abbreviations: NYHA New York Heart Association; OMT Optimal medical therapy

We conducted a model-based cost-effectiveness analysis which combined a decision tree and a Markov model to compare the clinical and economic consequences of the MitraClip combined with OMT versus OMT alone from the perspective of the SHI (see Fig. 1). The cost-effectiveness was expressed by the incremental cost-effectiveness ratio (ICER) which was calculated by dividing the differences in costs and QALYs and those in costs and life years gained (LYG) between MitraClip/OMT and OMT alone. To reflect the long-term consequences of HF, a lifetime horizon with a cycle length of 1 month was applied in the model.

Clinically, the cost-effectiveness analysis was mainly based on the COAPT trial, a multicenter randomized-controlled trial including 614 patients with ischemic or non-ischemic cardiomyopathy at 78 sites in the US and Canada. In that study, MitraClip/OMT showed a statistically significant reduction in ‘hospitalizations for HF at 12 months’ [Hazard Ratio (HR) = 0.53; *P* < 0.001] and, a significant reduction in mortality in favor of the MitraClip group compared with OMT (HR = 0.62; *P* < 0.001) [8].

To obtain further clinical and economic input data, literature searches using the Medline database were performed in January 2019 and updated throughout the model development (details are reported in the Appendix).

### Patients and model description

In the model, we evaluated a hypothetical cohort that was in line with patients’ characteristics of the COAPT trial. Patients had an ischemic or non-ischemic cardiomyopathy, a confirmed moderate-to-severe or severe secondary mitral regurgitation and, were categorized as symptomatic according to the New York Heart Association (NYHA) functional classes II, III, or IV. Patients entered into the model at mean age of 72 (SD 11.2) years and 64% of them were male [8].

The cohort started with a decision tree that divided patients by whether they received MitraClip/OMT or OMT alone (Fig. 1). Irrespective of treatment option received, patients then entered a Markov model consisting of five health states: the four NYHA functional classes and death. Based on data from the COAPT trial, patients moved between the NYHA classes. In all NYHA classes, patients could be admitted to the hospital due to HF [8] (with resulting costs and disutilities accordingly but without increasing the transition probability to higher NYHA states). The initial distribution of NYHA classes was calculated as an average and applied to both treatment options in the beginning of the first month. The effects of the device related complications for the MitraClip group at 12 months (3.4%) from the COAPT trial were included in the analysis, calculated as an average and distributed across all patients from the MitraClip group [8]. At the end of each month patients either remained in the same health state, experienced a HF-progress to a higher NYHA class or a HF-regress to lower NYHA class, or they died. The model was constructed and analyzed with Tree Age Pro 2020 (Tree Age Software LLC).

### Model inputs

#### Transition probabilities

To reflect both the short- and long-term effect of the MitraClip in the model, two different transition probabilities were applied for each NYHA class: one probability for the transition from baseline to 30 days and a second probability for the transition from 30 days to all subsequent cycles. For both groups in the COAPT trial, it could be observed that the patients NYHA status on average improved from baseline to 30 days whereas after 30 days the NYHA status gradually declined for all subsequent cycles. As the transition probabilities were not reported in the trial and were not made available (even on request), these had to be approximated from the 24-month follow-up lifetables by stepwise iteration. As a result of this approximation the majority of patients moved to the closest NYHA-group (e.g., NYHA I to NYHA II, NYHA IV to NYHA III) but skipping a class upwards or downwards (e.g., NYHA I to NYHA III or vice versa) was also possible. The monthly transition probabilities for all NYHA classes are shown in Table 1.

**Table 1:** Transition probabilities between NYHA states for both the MitraClip and Optimal Medical Therapy groups

	NYHA I	NYHA II	NYHA III	NYHA IV	HF death
MitraClip					
Baseline→30 days					
NYHA I	0.950	0.050	0.000	0.000	0.000
NYHA II	0.100	0.900	0.000	0.000	0.000
NYHA III	0.200	0.350	0.370	0.070	0.010
NYHA IV	0.140	0.250	0.270	0.320	0.020
30 days→all subsequent cycles					
NYHA I	0.960	0.040	0.000	0.000	0.000
NYHA II	0.005	0.945	0.050	0.000	0.000
NYHA III	0.000	0.025	0.895	0.070	0.010
NYHA IV	0.000	0.000	0.000	0.800	0.200
Optimal medical therapy					
Baseline→30 days					
NYHA I	0.950	0.030	0.020	0.000	0.000
NYHA II	0.100	0.870	0.030	0.000	0.000
NYHA III	0.050	0.170	0.700	0.060	0.020
NYHA IV	0.000	0.120	0.170	0.680	0.030
30 days→all subsequent cycles					
NYHA I	0.950	0.050	0.000	0.000	0.000
NYHA II	0.010	0.940	0.040	0.010	0.000
NYHA III	0.000	0.020	0.920	0.050	0.010
NYHA IV	0.000	0.000	0.000	0.750	0.250

*NYHA* New York Heart Association

Each cycle, the patients from both groups could be admitted to the hospital for HF based on hospitalization probabilities pulled from the COAPT trial. It was assumed that each patient could be hospitalized only once per cycle due to the data available from the COAPT trial [8].

#### Resource utilization and cost data

Data on resource utilization and cost were derived from German sources wherever possible (Table 2). Device costs, costs of hospitalization due to HF, and complication treatment costs were based on the German diagnosis-related groups (DRGs) 2020 [11] (OPS 5-35a.41 and 5-35a.60, i.e., the reimbursement for the procedure based on the average length of stay for the MitraClip). The costs of treatment for device related complications were calculated from the number of complications reported in the COAPT trial combined with the corresponding German DRG. Data on complications were obtained from the COAPT trial and included single leaflet device attachment (0.7%), device embolization (0.3%), left ventricular assistant device (LVAD) implantation (1.2%), cardiac transplantation (0.8%), and non-elective cardiovascular surgery (0.3%). Information about average costs for routine management of HF patients specific to each NYHA class (e.g., medical therapy, GP visits) was obtained from the German Competence Network Heart Failure (CNHF) Using a telemetric platform, the CNHF collects routine data from a large cohort of well HF patients based on standardized case report forms and centrally run data bases with automated revision and consistency checks [12, 13]. In line with the payer perspective of the analysis (i.e., German SHI), only direct medical costs were included and expressed in 2020 euros.

**Table 2:** Patient characteristics, NYHA class distribution, costs, utilities, and hospitalization rates

Variable	Base-case value	Range	References
Patient characteristics			
Age (years) (SD)	72.2 (11.2)	61–83.4	[8]
Male (%)	64	60–66	[8]
Initial NYHA class distribution (proportion in %)			
NYHA I	1.5		[8]
NYHA II	39.05		[8]
NYHA III	52.50		[8]
NYHA IV	8.30		[8]
Cost data (€)			
MitraClip device cost	32,434		[11]
Monthly cost routine management HF (SD)			
NYHA I	48	34–67	[12]
NYHA II	84	60–118	[12]
NYHA III	83	59–117	[12]
NYHA IV	90	64–126	[12]
Average cost of complications per patient (SD)	2530	1518–3542	[8, 11]
Hospitalization cost	3272		[11]
Monthly additional healthcare costs 65–85	807		[14]
Monthly additional healthcare costs 85 +	1912		[14]
Utility data (in %)			
NYHA states			
NYHA I	0.815	0.781–0.850	[15]
NYHA II	0.720	0.693–0.749	[15]
NYHA III	0.590	0.551–0.629	[15]
NYHA IV	0.508	0.412–0.605	[15]
One-month disutility for MitraClip procedure	0.043	0.034–0.051	[16]
One-month disutility for HF hospitalization	0.064	0.038–0.090	[17]
Average utility decrement due to complications	0.005	0.003–0.007	[17]
Hospitalization rates			
MitraClip			
NYHA I	0.0262	0.0231–0.0298	[8]
NYHA II	0.0262	0.0231–0.0298	[8]
NYHA III	0.0330	0.0295–0.0370	[8]
NYHA IV	0.0085	0.0068–0.0106	[8]
Optimal medical therapy			
NYHA I	0.0338	0.0302–0.0378	[8]
NYHA II	0.0338	0.0302–0.0378	[8]
NYHA III	0.0668	0.0617–0.0723	[8]
NYHA IV	0.0169	0.0144–0.0198	[8]

*NYHA* New York Heart Association; *HF* Heart failure; *SD* standard deviation

#### Utility data

Utilities reflect patients’ preferences for a specific health state and are used to estimate the patients’ quality of life. Patients in both treatment options accrued utility values regardless of the treatment received. Since no specific German utility data on NYHA classes were available, utility values were taken from the CARE-HF trial [18], a multicenter, international, randomized trial enrolling 813 patients from 82 European centers with HF. The trial compared pharmacologic therapy alone with a combination of pharmacologic therapy and cardiac resynchronization. Utilities were calculated using EuroQoL EQ-5D utility scores at different time measurement points in combination with Time Trade-Off (TTO) based patient preferences from the UK.

Because the use of the MitraClip is expected to affect a patient’s quality of life, a slight utility decrement of − 0.043 was assumed for patients in the device group for the first cycle (similar to that of a percutaneous coronary intervention [16]). In addition, patients in both arms were assigned a reduced quality of life as a result of complications related to the device implantation (− 0.004762) and hospitalization (− 0.064).

Data on costs and utilities were discounted using a discount rate of 3% yearly in line with the German Institute for Quality and Efficiency in Health Care (IQWiG) [19]. All model inputs are listed in Table 2.

### Sensitivity analyses

To assess the impact of varying the input parameters on the model results, one-way deterministic sensitivity analyses (DSA) were performed for all model parameters. To test the influence of simultaneous variation of input parameters on the model results, a probabilistic sensitivity analysis (PSA) was performed using a priori-defined variable distributions (e.g., beta distributions for probabilities, rates, and utility values, gamma distributions for costs). Because costs due to hospitalization and the MitraClip costs did not vary, none were tested in the PSA. We presented the results of the DSA on a tornado diagram and the results of the PSA in a cost-effectiveness acceptability curve [20].

In addition to the base-case analysis over a life-time horizon, a supplementary analysis was limited to the 24-month follow-up of the study. As recommended by the IQWiG [19], the health-care costs for additional life years gained were excluded from the base-case analysis but included in a sensitivity analysis. Further, a cost-effectiveness threshold analysis was performed for the device costs of the MitraClip.

### Model validation

To check how well our model represents chronic HF and whether it is appropriate to evaluate our main question we used several validation approaches [21]. To ensure sufficient consistency for the course of the model population with that of the COAPT trial, validation was made by verifying that at all times of the follow-up (0, 30 days, 6 months, …) the number of patients in the NYHA states was similar. We consulted experts on the adequacy of input data and the structure of the model (face validity). Technical accuracy was checked regarding data entry and potential programming errors (computerized model validation). For cross model validation, we assessed the extent to which our models came to different conclusions than published results of the COAPT-trial-based cost-effectiveness analysis [9] as well as a cost-effectiveness analysis based on the earlier EVEREST II HRS and REALISM trials [22].

## Results

### Base-case analysis

Over the life-time horizon, the total cost per patient of the MitraClip was € 43,152, whereas the cost of the OMT group amounted to € 9264. The MitraClip resulted in 0.57 additional QALYs compared with OMT (2.5 QALYs for MitraClip vs. 1.93 QALYs for OMT). In terms of life years gained, the MitraClip group gained 3.68 life years vs. 2.88 life years for the OMT group.

In comparison with OMT alone, the MitraClip resulted in an ICER of € 59,728/QALY (costs per incremental life years gained € 42,360). The results are displayed in Table 3.

**Table 3:** Results of the base-case cost-effectiveness analysis

Therapy option	Cost (€)	Incremental cost (€)	LYG	Incremental LYG	ICER (€/LYG)	Costs (€/LYG)	QALY gained	Incremental QALY gained	ICER (€/QALY)
Optimal medical therapy	9264		2.88			3195	1.93		
MitraClip	43,152	33,888	3.68	0.80	42,360	11,663	2.50	0.57	59,728

*LYG* Life years gained; *ICER* Incremental cost-effectiveness ratio; *QALY* Quality adjusted life year

### Sensitivity analyses

The results of the one-way DSA were most sensitive to the probability of hospitalization and the transition probabilities between the NYHA classes, especially for the transition between NYHA class II and III (Supplementary Fig. S4 in the Appendix). In contrast, varying the parameters for costs and utilities did not affect the cost-effectiveness (Fig. 4 in the Appendix).

**Fig. 2:**
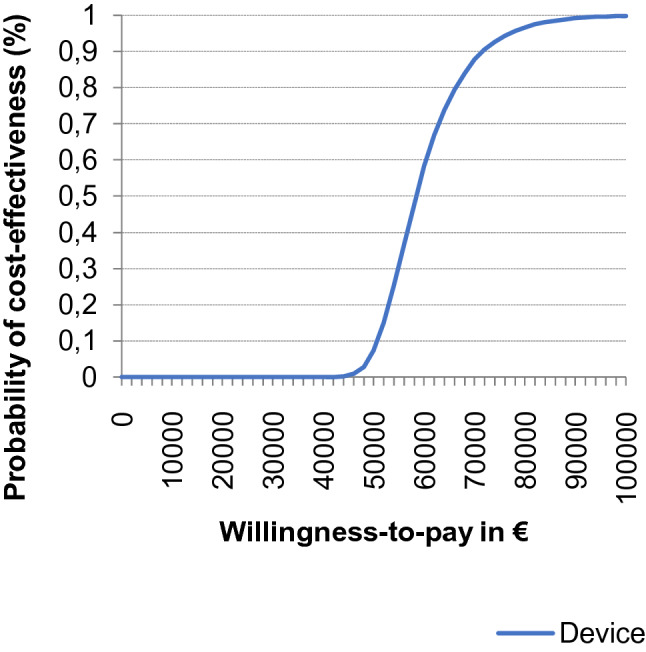
Cost-Effectiveness Acceptability Curve for the MitraClip. The figure shows the percentage of iterations where the MitraClip therapy is cost-effective at a specific willingness-to-pay threshold from the probabilistic sensitivity analysis (Monte Carlo simulation with 10 000 iterations)

In the PSA, at a willingness-to-pay threshold of € 60,000/QALY the MitraClip had a 58% probability of being cost-effective compared with OMT only, which increased to 88% at a threshold of € 70,000/QALY (Fig. 2).

**Fig. 3:**
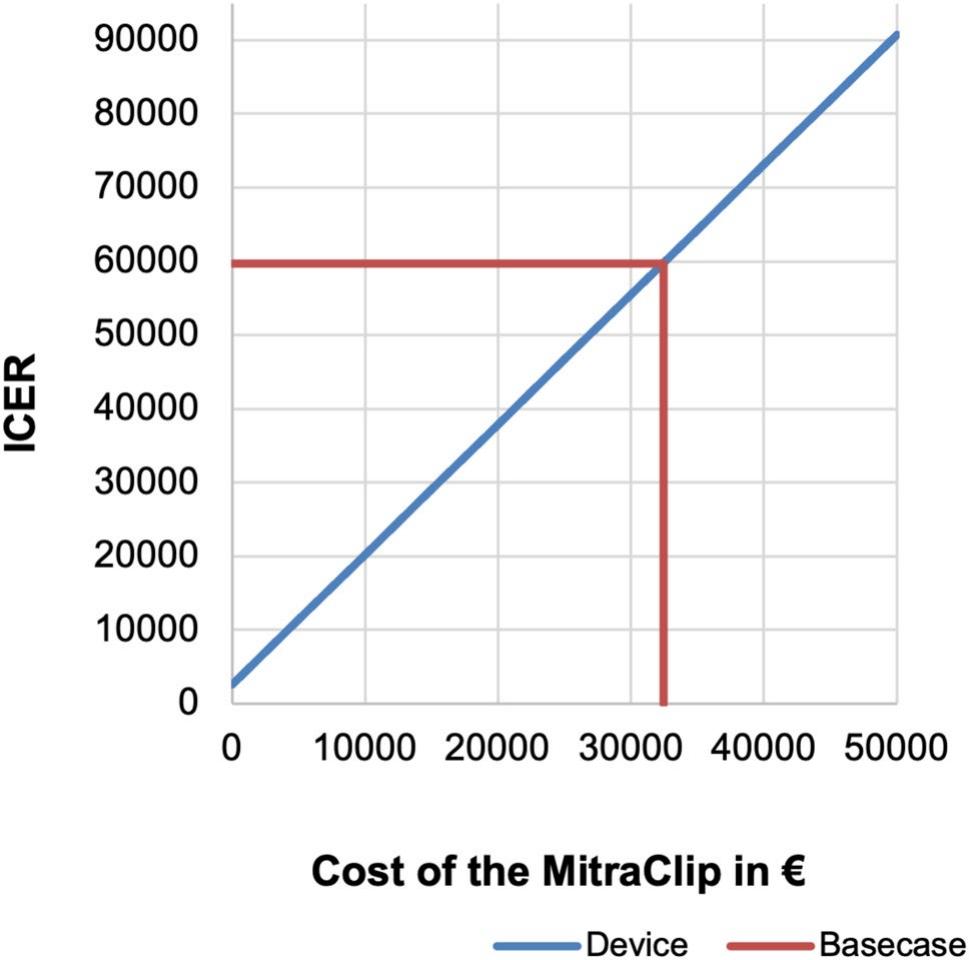
Threshold analysis for the Cost of the MitraClip: The figure shows the effect of varying the cost of the MitraClip on the incremental cost-effectiveness ratio (ICER). For example, at a cost of € 30 000 for the diagnosisrelated groups of the MitraClip, the ICER for the MitraClip strategy would be € 55 439.

An additional threshold analysis revealed that price reductions of the MitraClip would have a positive impact on the cost-effectiveness. A reduction of the MitraClip device cost from € 32,434 to € 25,000 would reduce the ICER of the MitraClip to € 46,626/QALY, whereas a reduction from 32,434 to € 20,000 would reduce the ICER of the MitraClip to € 37,814/QALY (Fig. 3).

In the analysis reflecting a shorter time horizon (e.g., 24 months), the MitraClip strategy resulted in an additional cost of € 33,945 for 0.20 additional incremental life years/ QALYs gained, leading to an ICER of € 199,400/QALY. Inclusion of the cost of the additional life years gained increased the cost-effectiveness ratio of MitraClip compared with OMT to € 73,468/QALY.

### Model validation

The validation of the model showed that the model could precisely predict all-cause mortality in both groups for the 2-year follow-up period in comparison with the trial data from the COAPT trial. On a population-basis at all times of the follow-up (0, 30 days, 6 months, …) the model was consistent with the NYHA class distribution observed in the COAPT trial. In addition, the model outcomes were judged as realistic by a clinical expert.

The cross-validation with results from previous cost-effectiveness studies showed that the results from our model were consistent with one analysis from the American COAPT trial [9] (ICER of $55,600/QALY). Compared with other analyses from different countries, the results of our analysis were less cost-effective, ranging from € 15,800 to € 35,200/QALY [9, 10, 23]. While the incremental costs were similar in all analyses, the number of utilities gained by the MitraClip was higher (between 1.1 and 1.4 compared to 0.6 in our analysis) [9, 10, 23].

### Discussion

This modeling study is a first step in evaluating the MitraClip in patients with heart failure and secondary mitral regurgitation in the German context.

The results of our cost-effectiveness study raise the question what additional costs would be appropriate for a gain of QALY/life years in Germany. Commonly used decision rules for new medical devices include thresholds based on income per capita [24]. Based on the threshold suggested by the World Health Organization (WHO) of a maximum of three times the national annual gross domestic product (GDP) per capita (€ 40,048 per Capita in Germany in 2020) [25] the MitraClip would represent cost-effective value in the German context with a value of around one and a half times the German GDP per capita. In addition to the threshold approach, new interventions can be benchmarked in comparison with an intervention that has already been adopted in the target country. In this approach, again a threshold is applied but—unlike the thresholds based on per capita GDP—the threshold is established by a retrospective analysis of existing practice [26]. However, for heart failure and secondary mitral regurgitation such a threshold is not available for Germany. Moreover, as neither concept is established in Germany, the final judgement remains the responsibility of the decision-maker (i.e., the SHI).

Compared with cost-effectiveness analyses from Canada [27], Japan [22], and the United Kingdom (UK) [8], the results of our analysis were less cost-effective (i.e., about twice as much). While the total incremental costs were similar in all analyses, the difference was mainly due to the number of utilities gained by the MitraClip. The analyses from Canada and Japan were based on clinical data from the EVEREST II HRS trial which showed a larger clinical benefit for the MitraClip group. However, the EVEREST II HRS trial was based on observational data on patients who were older (77 years vs. 72 years in the COAP trial in the intervention groups), had a larger proportion of NYHA III/IV patients (90 vs. 57%), and were at higher risk of mortality within 30 days (18 vs. 8%) [28]. The analysis from the UK was based on the COAPT trial (just like our analysis). However, for calculating the treatment effect, an extrapolation beyond the 2-year trial period was undertaken under the assumption that the NYHA-mix would remain constant beyond this period [23]. This assumption might have increased the gain of QALY due to the MitraClip.

With respect to the application of efficacy data in a cost-effectiveness model, even the results of randomized-controlled trials differed. Whereas the COAPT trial demonstrated a reduction in hospitalization rates and all-cause mortality, the MITRA-FR, a phase 3, multicenter, randomized, open-label, controlled trial conducted in France showed different results. In detail, no significant differences with regard to all-cause mortality for the MitraClip vs. OMT alone were observed. In addition, the rate of unplanned hospitalizations (48.7% (74 of 152 patients) for the MitraClip group and 47.4% (72 of 152 patients) for the OMT group) were similar between the groups [29]. These differences could be attributed to various causes such as patient selection and a more optimized medical therapy in the COAPT trial [30]. Therefore, a cost-effectiveness analysis based on the MITRA-FR is expected to result in a more unfavorable cost-effectiveness ratio.

Because of these contradicting results, the question of identifying those patients who may benefit most from the MitraClip has been raised. Some authors identified differences in the inclusion criteria of the two trials and argued that the effectiveness of the MitraClip may depend on a more targeted patient selection [30]. In comparison with the MITRA-FR trial, the COAPT trial recruited patients with a higher severity of mitral regurgitation and a smaller left ventricular size. Applying the same classification criteria for mitral regurgitation severity to both studies, only 16% of MITRA-FR patients but 41% of COAPT patients had severe mitral regurgitation, defined by EROA(Effective regurgitant orifice area) ≥ 40 mm^2^ [30].

Considering the results of both trials, patients with extensive left ventricular (LV) dilatation may have a smaller benefit from the MitraClip procedure because the HF is predominantly caused by the underlying cardiomyopathy rather than the valvular disease. In contrast, patients with less LV dilatation (LVESD ≤ 70 mm) and with a moderate-to-severe degree of mitral regurgitation (≥ 30 mm^2^ and a regurgitant volume (RV) ≥ 45 mL)—which corresponds to the target population reflected in our analysis—benefit from the reduction of mitral regurgitation and thus from the MitraClip procedure [30]. Concerning the applicability of the COAPT-trial data to the German setting, a real-world cohort in Germany showed that half of the patients undergoing the MitraClip procedure had baseline characteristics similar to those of the COAPT trial. Furthermore, these patients showed a substantially more favorable outcome than those without COAPT-like characteristics [31]. Therefore, for the cost-effectiveness of the MitraClip procedure this suggests that selecting patients with a high degree of mitral regurgitation and relatively preserved LV function is essential to achieve clinical outcomes at acceptable costs.

A threshold sensitivity analysis of the cost for the MitraClip device from the perspective of the German statutory insurance revealed that a price reduction of the MitraClip could improve its cost-effectiveness. In the future, commercial competition between manufacturers may result in a reduction of device costs and (in the long-term) a reduction in the DRG and OPS of the MitraClip. This could alter the results in favor of the MitraClip compared to OMT only.

Our modeling study has several limitations: first, because to date there are no utility values for German HF patients reported in the literature, our study was based on utility values from the CARE-HF trial. Because utility values reflect the cultural values and beliefs of the country where the data were collected [23, 32] these are of limited representativeness for German patients with HF. However, TTO base value sets for Germany and the UK are very similar according to the literature [32].

Second, as most of our clinical data, especially the transition and hospitalization probabilities, were based on the results of the COAPT trial the same limitations reported in the trial apply to our model [8]. In particular, the probabilities for the patient’s transitions between NYHA states were not reported in the trial and, data were not made available. For this reason, the approximation of the transition probabilities in our model may have resulted in slight deviations from the real transition probabilities between NYHA states. However, validation was made by verifying that on a population-basis at all times of the follow-up (0, 30 days, 6 months, …) the model population was consistent with the NYHA class distribution observed in the COAPT trial.

Third, the transition probabilities, particularly those between the NYHA states 2 and 3, were among the most sensitive parameters altering the model outcomes (in line with other economic evaluations for the MitraClip). Because utility decreases with higher NYHA states (I–II: − 0.095, II–III: − 0.13, and III–IV: − 0.082) a higher probability of moving from NYHA state I to II, II to III, and III to IV would reduce the cost-effectiveness ratio of the MitraClip as it would reduce the patient’s life expectancy [22, 27].

Fourth, for the transition between the NYHA states, it was assumed that patients moved between NYHA states according to the transition probabilities derived from the COAPT trial irrespective of their hospitalization status. This simplification was necessary as no separate data for the transition between NYHA states depending on the hospitalization status was reported in the COAPT trial. One might argue that a hospitalized patient is more likely to jump groups (i.e., NYHA I or II to NYHA IV). However, including this clinical scenario in our model would not alter the results on a cohort level as the distribution of patients in NYHA states at specific time points was in line with what was reported in the COAPT trial.

Fifth, since data on efficacy are based on the COAPT trial, the results of our study are only applicable to patients similar to those in the COAPT trial [8]. However, rates of mortality and hospitalization obtained from the German TRAMI registry were similar to the rates reported in the COAP trial (e.g., 24-month mortality rate of 29.1% in the COAPT trial vs. 31.9% in the TRAMI registry). Thus, the external validity of the trial data was considered to be acceptable [8, 33].

Finally, a further limitation of our study is that the transition probabilities of the model were based on the 24-month-follow-up of the COAPT trial but applied to a life-time horizon. However, our sensitivity analysis with a 24-month model horizon showed similar results for the MitraClip compared to the life-time horizon (i.e., the MitraClip was slightly less cost-effective).

As HF incidence in Germany is rising with an increasing need of health-care resources, more and precise long-term data especially for the transitions between NYHA states for the MitraClip procedure would be useful to re-evaluate the MitraClip for cardiac treatment in Germany [30]. Furthermore, specific utilities for the German patients with HF would be useful to precisely assess the impact of health technologies in the German context.

## Conclusions

In the German context, the MitraClip can be a cost-effective intervention for the treatment of secondary mitral regurgitation in patients with HF. However, compared with previous cost-effectiveness studies based on earlier and non-randomized trial data, our analysis suggests that the MitraClip is less cost-effective than previously evaluated. Recent clinical data indicates that patient recruitment may be key to identify the right patients for the procedure, i.e., patients with preserved LV function and a high degree of mitral regurgitation benefit most from the procedure. In the future, as the indication for the MitraClip device evolves and data on both the likelihood of changes between NYHA states and long-term effects becomes available, additional economic evaluation should be performed on a more robust data basis.

## Supplementary Information

Below is the link to the electronic supplementary material.

Supplementary file1 (DOCX 23 KB)
