# Supplementary material for: Cost-effectiveness of the MitraClip device in German heart failure patients with secondary mitral regurgitation
Source: Eur J Health Econ. 2022 May 27;24(3):349–58. doi: 10.1007/s10198-022-01476-4 (PMC10060324; doi:10.1007/s10198-022-01476-4)
Supplement: Supplementary file 1 — Supplementary file1 (DOCX 23 KB) [file 10198_2022_1476_MOESM1_ESM.docx]

**Supporting information**

**Article title:** Cost-effectiveness of the MitraClip device in German heart failure patients with secondary mitral regurgitation

**Journal**:

**Authors**:

- Bent Estler
- Yana Seleznova
- Arim Shukri
- Stephanie Stock
- Dirk Müller

Affiliation and e-mail address of the corresponding author:

Institute for Health Economics and Clinical Epidemiology, the University Hospital of Cologne (AöR)

[bestler@smail.uni-koeln.de](mailto:bestler@smail.uni-koeln.de)

Contents

1. Literature research
2. Figure 4: Tornado diagram of the one-way sensitivity analysis

# Literature research

A literature research was performed in January 2019 in Medline, via PubMed, in order to obtain data on event rates / treatment efficacy, usual treatment patterns, and utilities (which should be based either on the time trade off or the standard gamble method). If more than one source was available, data reflecting a higher level of evidence and sources reflecting the German context more appropriately was preferred.

Articles reflecting data on costs were assessed using the following criteria:

1. Description of settings, locations, and relevant dates, including periods of recruitment,
2. Follow up, and data collection
3. Detail of sources of data, details of methods of assessment (measurement)
4. Description of efforts to address potential sources of bias
5. Explanation how quantitative variables were handled in the analyses.

Before including data in the model, clinical experts were asked regarding the suitability of data in the specific clinical context.

Key words: “functional mitral regurgitation”, “heart failure”, “MitraClip”, “costs and cost analysis”, “hospitalization””mortality”, utilities, preferences, and resource use.

**Figure 4**: **Tornado diagram of the one-way sensitivity analysis**

The upper limit varied in the deterministic sensitivity analysis for each parameter is shown in grey whereas the lower limit for the probabilities is shown in black. The most sensitive parameters in the deterministic sensitivity analysis were the transition probabilities between NYHA state 2 to NYHA state 3 for both the device and the control group.
